# A century of Morita therapy: What has and has not changed

**DOI:** 10.1111/appy.12511

**Published:** 2022-04-10

**Authors:** Mitsuhiro Nakamura, Hidehito Niimura, Kenji Kitanishi

**Affiliations:** ^1^ Department of Psychiatry Yokohama Camellia Hospital Yokohama Kanagawa Japan; ^2^ Shinano Mental Clinic Yokohama Kanagawa Japan; ^3^ Faculty of Human Science Toyoeiwa University Yokohama Kanagawa Japan; ^4^ Department of Neuropsychiatry Keio University School of Medicine Tokyo Japan; ^5^ Morita Therapy Institute Tokyo Japan; ^6^ Kitanishi Clinic Tokyo Japan

**Keywords:** Morita therpay, outpatient, psychotherapy

## Abstract

We review the history of Morita therapy (MT), which has existed for over 100 years, and examine what has changed over that period and what has not. Classic MT, which was dependent on a highly strict therapeutic approach, gradually lost its pre‐eminence, but at the same time, the fundamental theory of MT was refined. This theory came to be applied to current outpatient MT and adapted to inpatient MT. As MT was refined, a standard training system for therapists was established, adaptations to modern conditions were made and expanded, and comparisons to and dialogs with other psychotherapeutic concepts such as mindfulness became possible. To better evaluate MT, further work should be conducted on its effectiveness of from a clinical epidemiological perspective.

## 1 INTRODUCTION

Morita therapy (MT) is a psychotherapy for neurosis developed by Shoma Morita in 1919 (Fujita, 1986; Goddard, 1991; Han et al., 2021; Ishiyama, 1986b; Iwai & Reynolds, 1970; Jacobson & Berenberg, 1952; Kawai & Kondo, 1960; Kitanishi & Mori, 1995; Kondo, 1953; Kora, 1965; Nakamura & Kitanishi, 2019; Ohara & Reynolds, 1968; Yamadera, 2016). It was long considered to be uniquely suited to Japan and Japanese culture, and it was only introduced to the West in the 1950s and 1960s (Jacobson & Berenberg, 1952; Kondo, 1953; Kora, 1965; Kora & Sato, 1958). At that time, classic MT was largely discussed in relation to Zen and Japanese culture. Reynolds et al. later showed that MT is not a psychotherapy to be understood only in relation to Japanese culture or Zen but was based on human nature and has wide application to the West, where MT long had a reputation of being overly rigid and structured (Iwai & Reynolds, 1970; Reynolds, 1976; Walton, 1985). However, in Japan, the number of facilities in which classic inpatient MT can be practiced has decreased, and MT had transformed into a dialog‐based psychotherapy by the 1990s (Kitanishi, 2014; Kitanishi & Mori, 1995; Nakamura & Kitanishi, 2019; Ohara & Reynolds, 1968; Sugg et al., 2017). This paper reviews the history of MT in Japan over the past 100 years and discusses themes related to the practice of MT today in relation to the expanding applications of MT, its relationship to mindfulness and ACT, and its effectiveness.

## 2 WHAT HAS CHANGED: THERAPEUTIC SYSTEM

### 2.1 Classic inpatient MT (as practiced by Shoma Morita)

An outstanding feature of early MT was that treatment was performed at Morita's own home. His family, especially his wife, acted as assistant therapists, and the treatment area had a familial and accepting atmosphere (Fujita, 1986; Iwai & Reynolds, 1970; Kitanishi & Mori, 1995; Kora, 1965; Morita et al., 1998; Reynolds, 1976; Suzuki & Suzuki, 1981).

However, early MT was stricter than modern outpatient MT (Walton, 1985). Morita divides MT treatment into four different stages: (1) isolation rest, wherein the patient lies on a bed and is deprived of stimulation; (2) light monotonous work, performed without verbalization with assigned diary writings that begin a written therapeutic dialog between the patient and therapist; (3) labor‐intensive work and assigned diary writings; and (4) social integration including errands outside the treatment area (Fujita, 1986; Iwai & Reynolds, 1970; Kora, 1965; Morita et al., 1998; Reynolds, 1976). Morita's treatment was a combination of rest and discipline (Kitanishi & Mori, 1995). During inpatient MT, the therapist maintains the *fumon*,* or non‐inquiry, principle, encouraging patients to carry out daily activities in spite of their symptoms as part of the therapy (Fujita, 1986; Kitanishi & Mori, 1995; Morita et al., 1998; Sugg et al., 2020).

Morita theorized that psychiatric symptoms were the result of a hypochondriasis temperament*, coupled with opportunity and the cause of illness (psychiatric interaction*) (Fujita, 1986; Kitanishi & Mori, 1995; Morita et al., 1998). Patients with the hypochondriasis temperament* consider their natural emotional response to an event (opportunity) in the outside world to be inimical to their survival and adaptation, and they fixate on that response (Fujita, 1986; Kitanishi & Mori, 1995; Morita et al., 1998). Their emotional response is then felt even more sharply and intensely, attracting their attention to an even greater degree (psychiatric interaction*). This vicious cycle, passing from sensation to attention and back again, leads to the formation of and fixation on symptoms (Kitanishi, 2005; Kitanishi & Mori, 1995; Kondo & Kitanishi, 2014; Ogawa, 2013; Sugg et al., 2020). As his treatments became more complex, Morita focused on eradicating the conflict between ideals and reality, paying closer attention to the patient's desire to live (perfectly) (Kitanishi, 2005; Morita et al., 1998; Sugg et al., 2020). The desire to live plays two roles: (1) amidst suffering, the desire to live works to eliminate anxiety or fear, but (2) it can also serve to change a person's desires. Morita believed that this would naturally give way to realizing one's true self (Fujita, 1986; Kitanishi, 2014; Morita et al., 1998). He referred to this state as *arugamama*,* or accepting reality as it is, becoming a therapeutic goal (Davis et al., 1972; Fujita, 1986; Kitanishi, 2005; Kitanishi & Mori, 1995; Kora, 1965; Morita et al., 1998; Ogawa, 2013; Reynolds, 1976; Sugg et al., 2020; Tateno, 2016).

### 2.2 After Morita's death

During Morita's life, his treatment remained unknown in the Western world. MT was first discussed in the Western psychology literature in the 1950s and 60s (Jacobson & Berenberg, 1952; Kondo, 1953; Kora, 1965). Western researchers brought renewed attention to Eastern psychotherapy in the 1970s and 80s, publishing papers and books on MT (Iwai & Reynolds, 1970; Reynolds, 1976). In China, MT was introduced in 1990 and spread throughout the country, and in 1997, the China Society for MT was established. In 1998, Morita's *Morita Therapy and the True Nature of Anxiety‐Based Disorders* was translated (Morita et al., 1998). In the 21st century, many works in English have been published on MT (LeVine, 2020; Mashima, 1992). Its effectiveness has been widely assessed from a clinical epidemiological view (Feng et al., 2020; Jia et al., 2018; Li & He, 2008; Sugg et al., 2017; Sugg et al., 2018).

In Japan, after Morita's death, Usa and Suzuki, who were close to Shoma Morita, opened Sansei Hospital in Kyoto and Suzuki Hospital in Tokyo and practiced MT (Davis et al., 1972; Kurokawa, 2006; Miura & Usa, 1970; Suzuki & Suzuki, 1981)). The Jikei University Hospital, where Morita was the first psychiatry professor, started inpatient MT, and several successors of Morita later opened hospitals to perform inpatient MT. Thus, there are numerous institutes in which inpatient MT was performed throughout Japan, and The Japan Morita Therapy Society was established in 1983 (Kitanishi & Mori, 1995; Reynolds, 1976).

However, the practice of classic MT declined from the late 1980s. Hospitals performing classic MT closed from that period, including Sansei Hospital, which closed in 2014 (Usa, 2003). The Jikei University Hospital, the last hospital in Japan to perform classical MT, stopped practicing it in 2020.

Classic MT places a large burden on the practitioner, requiring 24‐h availability in a family‐like environment, and few young therapists invested the effort into studying it; it was also considered a burden on the national insurance system (Kitanishi, 2005; Kitanishi et al., 2002; Kitanishi & Mori, 1995; Reynolds, 1976). Additionally, no systematic, institutionalized courses were available in classic MT. Finally, even those patients who did seek out MT often preferred dialog‐based therapy to the inpatient version (Kitanishi et al., 2002; Kitanishi & Mori, 1995; Ohara & Reynolds, 1968; Reynolds, 1976).

## 3 WHAT HAS NOT CHANGED: THEORY CLARIFIED BY CHANGES IN THE THERAPEUTIC SYSTEM

Table 1 shows what has and what has not changed in the 100‐year history of MT.

**TABLE 1:** What has and has not changed in the 100‐year history of Morita therapy (MT)

Year	Therapeutic system (what has changed)	Fundamental theory (what has not changed)	Surrounding situation
1919–1940s	*Classic inpatient MT* Highly structured with four stages Fumon principle and action‐center model Paternal therapist Family‐like environment; therapy conducted in Morita's home	Human nature understanding based on Oriental natural theory Desire to live and fear of death Cause of neurosis; hypochondriac temperament + opportunity (inadaptability anxiety) + psychiatric interaction Toraware (1) Vicious cycle theory (psychiatric interaction) (2) Contradiction by ideas (“should” thinking) Therapeutic goal; arugamama state	Patients' improvement was determined by therapist's own judgment rather than by a quantitative scale
1950–1970s	Morita's successors opened inpatient MT hospitals Therapeutic techniques, processes, and therapist–patient relationships were investigated		Japanese Society for MT established Introduced to the Western world and China
1980–1990s	Classic inpatient MT hospitals decreased *Modified inpatient MT* Inpatient MT system was conducted in the MT center of university hospitals or general psychiatric wards. *Outpatient MT* Dialog‐based individual psychotherapy Affection‐center model Refinement of vicious cycle theory Dealing with anxiety and fear as being paired with a desire to live		Expansion of applications Guidelines for outpatient MT were published Training system was established China Society for MT was established
2000s–	Classical inpatient hospitals closed		Discussion on comparison to other contemporary psychotherapies Clinical epidemiological effectiveness measurement study

### 3.1 Reevaluation of the theory and techniques of MT

In the latter half of the 20th century, a reevaluation of MT theory and its techniques took place. The transformation of the classic MT system has clarified its unchanging essence.

Although classic inpatient MT had declined, various types of modified inpatient MT, which was based on refined MT theory, emerged. There was an emphasis on discipline and action (Feng et al., 2020; Jia et al., 2018; Li & He, 2008; Sugg et al., 2017; Sugg et al., 2018), relief and rest in a familial environment (LeVine, 2017; LeVine, 2020), and the patient–therapist relationship in a general psychiatric ward (Mashima, 1992; Ogawa, 2013; Tateno, 2016). The Japanese Society for MT established a training system in 1998, replacing the apprenticeship system (Reynolds, 1976).

### 3.2 Understanding human nature through Eastern theory of nature

Morita called MT natural therapy, but it was later found to be based on an Eastern theory of nature and human understanding (Fujita, 1986; Kitanishi, 2005; Kondo & Kitanishi, 2014; Kora, 1965; Morita et al., 1998; Shinfuku & Kitanishi, 2010; Sugg et al., 2020).

Morita viewed human suffering in a circular view of nature rather than linear causality. MT places nature at the center. In MT, unpleasant physical and emotional reactions like fear, anxiety, and obsession are considered natural. Instead, perceptions, responses, and attention paid to these natural reactions were the problem. Psychopathology is the result of a vicious cycle. This founds the therapeutic theory and goals (Fujita, 1986; Kitanishi, 2005; Kondo & Kitanishi, 2014; Kora, 1965; Morita et al., 1998).

### 3.3 Vicious cycle theory

A person with a natural tendency to fall easily into anxious states brought about through internal or external stimulation (the latter is what Morita means by “opportunity”) experiences emotional reactions that could nevertheless occur in anyone. However, considering this natural reaction to be negative (contradiction by ideas*), the person comes to narrowly focus their attention on this reaction (Fujita, 1986; Kitanishi & Mori, 1995; Reynolds, 1976), which intensifies the emotional reaction, attracting the attention to it even more acutely (psychic interaction*) and creating a vicious cycle, or *toraware*.* Instead of searching for the original cause of the condition, one should dissolve the vicious cycle (Kitanishi, 2005; Nakamura & Kitanishi, 2019; Reynolds, 1976).

### 3.4 Contradiction by ideas* (“should” thinking)

In Buddhism, suffering is understood as the inability to control things according to our will or encountering something that does not follow our wishes. We consider our bodies, minds, and all other phenomena as ours, and we suffer when we seek to control them (LeVine, 2017; Shinfuku & Kitanishi, 2010; Sugg et al., 2020).

Morita explained the suffering of all human beings using the notions of desire and fear (Chen, 1996; Fujita, 1986; Ishiyama, 1986a; Kora, 1995; LeVine, 2017; Morita et al., 1998; Nakamura & Kitanishi, 2019; Ogawa, 2013). He believed that desire caused fear, following the Buddhist understanding of suffering. The desire to live creates the fear of death, which is suffering. Thus, creating harmony between desire and fear (suffering) was important. For Morita, desire and fear were natural phenomena (Chen, 1996; Fujita, 1986; Ishiyama, 1986a; Kora, 1995; LeVine, 2017; Morita et al., 1998; Nakamura & Kitanishi, 2019; Ogawa, 2013) and the basic organization of human conflict was an opposition to nature or a conflict between reality and an ideal (Kitanishi, 2005; Shinfuku & Kitanishi, 2010; Sugg et al., 2020).

### 3.5 Fear and desire

Morita drew on Buddhist ideas of the centrality of desire and its non‐fulfillment in human suffering and fear (Fujita, 1986; Kondo & Kitanishi, 2014; Kora, 1965; Morita et al., 1998; Nakamura & Kitanishi, 2019; Shinfuku & Kitanishi, 2010). In Morita's understanding, the desire to live produces the fear of death, or suffering, and he hoped to harmonize these natural phenomena (Fujita, 1986; Ishiyama, 1986a; Nakamura & Kitanishi, 2019; Ogawa, 2013). The conflict between nature and (logocentric) thinking was thus fundamental to humanity's conflicts (Fujita, 1986; Kitanishi, 2005).

### 3.6 Arugamama*: The therapeutic goal

*Arugamama*,* the therapeutic goal of MT, has two faces: the acceptance of emotions and fears and the desire to live. What is frightening simply is frightening, and this cannot be helped. It must be accepted and experienced. When patients feel this affliction fully, they become aware of their desire to live and able to demonstrate this as actions in the world. These two are closely related and dynamically form the patient's real life (Davis et al., 1972; Fujita, 1986; Kitanishi, 2005; Kora, 1965; Morita et al., 1998; Nagatsu, 2020; Ogawa, 2013; Reynolds, 1976; Sugg et al., 2020; Tateno, 2016).

## 4 WHAT HAS CHANGED: CLARIFICATION OF THERAPEUTIC TECHNIQUES IN OUTPATIENT MT

### 4.1 Sharing the vicious cycle between therapist and patient

Patients often consider themselves unable to do something for some hidden reason. However, in reality, their problem is generated from their relationship with their external environment, which includes others. By overcompensating with the issue they produce, they make only often excessive and futile efforts (Kitanishi, 2014; Nakamura & Kitanishi, 2019; Ogawa, 2013; Tateno, 2016).

### 4.2 How to become aware of and correct “should” thinking

Patients maladjusted to their living environment and with inadaptability anxiety* are overwhelmed and passive in their environment. Consequently, they expand their sense of self, which only strengthens “should” thinking (Fujita, 1986; Kitanishi, 2014; Kora, 1965; Morita et al., 1998; Nakamura & Kitanishi, 2019; Tateno, 2016). To recognize and reduce this “should” thinking, it is necessary to look back on the life history of the patient and sympathize with the patient's suffering, while also bringing the patient to understand the Morita perspective, where symptoms and suffering are inevitable and must be accepted (Kitanishi, 2014; Nakamura & Kitanishi, 2019; Tateno, 2016).

For example, patients with social phobia become attached to the idea that they “should” be relaxed and speak smoothly. Their belief that their anxiety “should” not be present shapes their symptoms, but in reality, anxiety and tension can be understood not only as perfectly natural but necessary (Chen, 2010; Ishiyama, 1983; Ishiyama, 1986b; Nakamura & Kitanishi, 2019; Tateno, 2016). Furthermore, patients who experience an expanding social life may apply their dogmatic expectations about how they should be or should not be not only to themselves but to others as well, becoming irritated with what they perceive to be other people's imperfections as a result. The therapist can increase the patient's awareness of his or her own self‐defeating patterns and offer instructions and encouragement to allow them to move their current dogmatic lifestyle to a more realistic one (Chen, 2010; Ishiyama, 1983; Ishiyama, 1986b; Kitanishi, 2014; Nakamura & Kitanishi, 2019; Tateno, 2016).

In MT, therapists perform two interventions simultaneously: (1) reducing “should” thinking and (2) informing patients what they can do. An intervention that is paired with lessening “should” thinking is an intervention that guides patients to consider what they can do. That is, patients are to be encouraged to feel all emotions in their life situations and continue to change their actions. The therapist can perform these two interventions simultaneously (Ishiyama, 1986a; Kitanishi, 2014; Nakamura & Kitanishi, 2019; Ogawa, 2013).

### 4.3 Developing techniques to address emotional experiences in life situations and promote actional change

In themselves, emotions have no value, positive or negative, and therapeutic dialog and diaries are used to build a therapeutic relationship that allows people to openly express all kinds of emotions and encourages them to take steps to move forward in their daily lives (Ishiyama, 1983; Ishiyama, 1986b; Kitanishi, 2014; Nakamura & Kitanishi, 2019; Ohara & Reynolds, 1968; Tateno, 2016). To do this, we pair emotional experience and actions and deal with them simultaneously. Specifically, this means: (1) experiencing emotions (symptoms) as they are, not fighting them (giving up trying to control them) but dealing with them in daily life and (2) taking transforming actions in the context of life experience (Ishiyama, 1986a; Kitanishi, 2014; Nakamura & Kitanishi, 2019; Ogawa, 2013; Tateno, 2016).

### 4.4 Dealing with both desire and fear simultaneously

**FIGURE 1:**
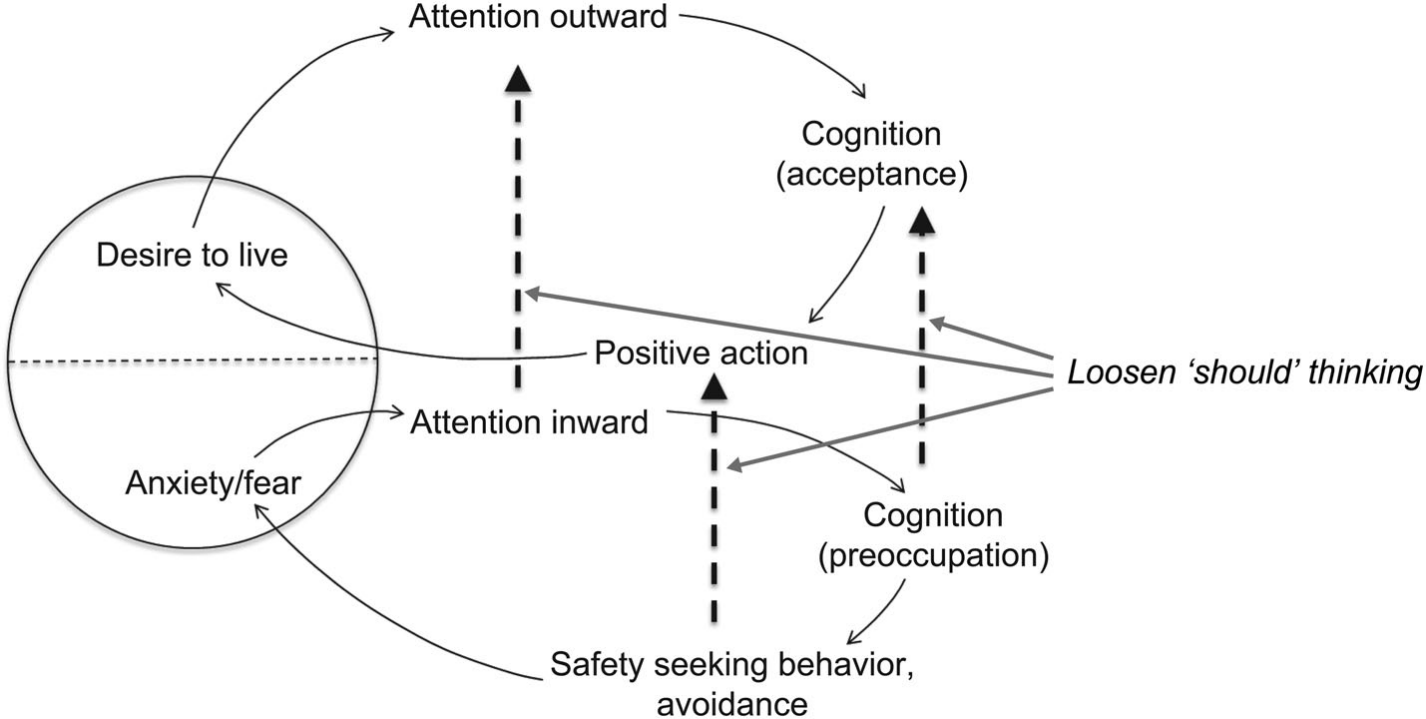
Illustration detailing the relationship between fear and desire, vicious cycle, and therapeutic intervention

Morita therapists view fear and anxiety as a single entity with two faces, one relating to feeling frightened and the other relating to the desire to live. Furthermore, MT considers that both anxiety and fear ultimately originate in the desire to live. MT emphasizes the positive aspect of fear (Fujita, 1986; Ishiyama, 1986a; Kora, 1965; Morita et al., 1998; Nakamura & Kitanishi, 2019; Ogawa, 2013; Shinfuku & Kitanishi, 2010) (Figure 1).

## 5 CURRENT DISCUSSION POINTS

### 5.1 Expansion of the applications of MT

Morita considered that anxiety that the mind and body might be pathological (hypochondriacal temperament*) to be a preparatory state for the generation of symptoms that would constitute an indication for MT (Fujita, 1986; Kitanishi & Mori, 1995; Morita et al., 1998). However, Kora interpreted this in a broader sense, and oriented therapy toward the anxiety of not being able to adapt to the environment in one's current state (inadaptability anxiety*) (Fujita, 1986; Kora, 1965; Kora, 1995). This kind of anxiety can appear in any condition and in any case, where people tend to have a “should” mentality (Chen, 2010; Ishiyama, 1983; Ishiyama, 1986b; Kitanishi, 2014; Nakamura & Kitanishi, 2019). Therefore, the techniques for dealing with fear and desire in MT can be applied to any condition, including anxiety disorders, mood disorders, and personality disorders described in the modern DSM‐5 (Chen, 2010; Ishiyama, 1983; Ishiyama, 1986b; Kitanishi, 2014; Nakamura & Kitanishi, 2019). That is, MT is widely applicable to modern patients who are unable to accept their suffering as it is and thus are unable to live as their authentic and natural self (Chen, 2010; Dehghan et al., 2021; Kitanishi, 2014; Nagatsu, 2020; Nakamura & Kitanishi, 2019).

### 5.2 Similarities and differences between arugamama* in MT and mindfulness

The similarities between mindfulness, which has attracted much attention in recent years, and the idea of arugamama*, which has a 100‐year history, has been discussed in the literature (Hayes, 2008; Hofmann, 2008; Nagatsu, 2020; Ogawa, 2013; Spates et al., 2011). Both are influenced by Zen and Buddhism, but there are significant differences between these two. Mindfulness adopts the main function of emotional regulation seen in Zen and Buddhism, removes the religious context, and incorporates it into a pre‐existing Western psychotherapeutic approach (cognitive behavioral therapy), which aims to objectify and control a patient's emotion. On the other hand, MT does not seek to objectify or control thought and emotion. In the concept of *arugamama*,* the “I” is also part of nature and in harmony with its surroundings (Hayes et al., 1999; Hofmann, 2008; Nagatsu, 2020; Ogawa, 2013; Tateno, 2016). Furthermore, in contrast to mindfulness, which accepts the body and mind as they are, the state of arugamama* contains goes beyond accepting the body, mind, and symptoms to seeking to fulfill the desire to live (Davis et al., 1972; Fujita, 1986; Hayes, 2008; Hofmann, 2008; Kitanishi, 2005; Kitanishi & Mori, 1995; Kora, 1965; Morita et al., 1998; Nagatsu, 2020; Ogawa, 2013; Reynolds, 1976; Sugg et al., 2020; Tateno, 2016).

### 5.3 Evaluation of MT's effectiveness from a clinical epidemiological view

In the 21st century, measuring the effectiveness of MT from a clinical epidemiological view has become an important task (Hayes, 2008; Sugg et al., 2018). Morita, Kora, and Suzuki used their own judgment to decide whether their patients had become better (Kitanishi & Mori, 1995; Kora, 1965; Morita et al., 1998). However, in recent years, it has become essential to have evaluations that establish the effectiveness of a treatment or change in symptoms through a validated scale (Hayes, 2008; Sugg et al., 2018).

In China meta‐analyses have been published for the treatment of schizophrenia and adult depression by MT in 2020 and 2018 (Feng et al., 2020; Jia et al., 2018; Li & He, 2008). Although their method was epidemiologically relevant, their paper only collected cases from China (Feng et al., 2020; Jia et al., 2018; Li & He, 2008). Furthermore, their version of MT was based on structured, action‐centered classic MT. As the authors stated in their paper, generalizing their results globally would be problematic (Feng et al., 2020; Jia et al., 2018; Li & He, 2008). In the United Kingdom, a pilot randomized control trial for depression using MT has been begun (Sugg et al., 2017; Sugg et al., 2018; Sugg et al., 2019; Sugg et al., 2020). However, in that context, MT was structured as a group‐based approach, meaning that it was partially classic MT (Sugg et al., 2018; Sugg et al., 2020). In Japan a committee has been founded to discuss how to measure the effectiveness of MT. In the future, for MT to be considered effective worldwide, we must assess its effects using epidemiological methods in every country.

## 6 CONCLUSION

The 100‐year history of MT makes clear that classic MT, which was dependent on a highly structured therapeutic system, has changed or fallen out of practice over the years. However, the essence of MT and its therapeutic techniques were formulated and formalized in the 1980s, and this has enabled the development of a training system for therapeutic techniques for MT. Contemporary discussions of MT relate to the differences between *arugamama* and mindfulness, the expansion of the applications of MT, and the need to determine the effectiveness of the therapy from a clinical epidemiological perspective.

Technical terms denoted in this manuscript with an asterisk (*) are explained in the Appendix.

## CONFLICT OF INTEREST

The authors declare no conflict of interest.

## Supporting information


**Appendix** S1: Supporting Information

## APPENDIX A

*Arugamama* (“as‐is” or “as it is”) is a concept that is opposed to *toraware* and the primary Morita therapeutic goal, in which one accepts reality as it is and responds accordingly. It is not a psychological state as such but the redirection of energy and attention to fulfill daily tasks.

Contradiction by ideas refers to thinking about what one might be or should be, as opposed to what one actually is. A person with a hypochondriacal temperament affected in this way will self‐evaluate his or her true self negatively. Such a person aspires to an ideal self‐image that is on a high plane and is inclined to “should” thinking. This aspiration puts the phenomenon of readily denying the fact of what one actually is into play.

*Fumon* (non‐inquiry/strategic inattention) is the central technique used in classic inpatient MT. In this approach, the therapist takes a non‐inquiry approach to the patient's subjective complaints and symptoms of anxiety to bring the patient to concentrate on meaningful behavior. Therapists implement *fumon* in response to patients' complaints to shift their attention away from their symptoms and toward purposeful action. Thus, therapists do not dwell on the symptoms or attempt to elucidate reasons for the suffering, beyond stressing the naturalness of all emotions, explaining how the vicious cycle functions for the individual, and highlighting the specific desires underlying fears.

A hypochondriacal temperament is an inclination to be oversensitive to psychological difficulties, physical sensations, and social interactions.

Inadaptability anxiety is Kora's in‐depth reinterpretation of Morita's concept of the hypochondriacal temperament. Kora presents this concept as the mood in which an introvert feels himself or herself at a disadvantage in relation to his or her mental and physical state; in other words, this is the introvert's experience of the anxiety regarding his or her inadaptability to his or her environment.

Psychic interaction describes the case where commonplace sensations that were ignored in the past now take on a sudden new meaning, provoking an intense anxiety reaction that cannot be ignored. The mental process or the person's attention slips out of control of conscious intention, and he or she begins instead to concentrate his or her attention on sensations such as the heart's throbbing and palpitation and the shortness of the breath due to the sudden anxious reaction to the idea of having a weak heart and being prone to suffer a heart attack at any moment. This reaction arises from a judgment based on a hypochondriacal tendency or temperament. As a result, the receptiveness to such sensations becomes highly sensitized, and this tendency further intensifies sensations and lowers practical functionality. Attention becomes more and more focused on these sensations. This process is what Morita terms psychic interaction.

*Toraware* (preoccupation) is a mental fixation, mental attachment or blocked flow of attention and mental energy due to cognitive rigidity and preoccupations with certain aspects of physical and mental experience that restricts the peripheral awareness necessary to respond to current circumstances. In Morita therapeutic theory, *toraware* is composed of psychic interaction and ideational contradiction. Reducing *toraware* in Morita therapy is therefore one of the critical turning points in patients' therapeutic progress.
