# Supplementary material for: A century of Morita therapy: What has and has not changed
Source: Asia Pac Psychiatry. 2022 Apr 10;15(1):e12511. doi: 10.1111/appy.12511 (PMC10078264; doi:10.1111/appy.12511)
Supplement: Supplementary file 1 — Appendix S1: Supporting Information [file APPY-15-0-s001.docx]

Supplementary information

Fumon (non-inquiry/strategic inattention) is the central technique used in classic inpatient MT. In this approach, the therapist takes a non-inquiry approach to the patient’s subjective complaints and symptoms of anxiety to bring the patient to concentrate on meaningful behavior. Therapists implement fumon in response to patients’ complaints to shift their attention away from their symptoms and toward purposeful action. Thus, therapists do not dwell on the symptoms or attempt to elucidate reasons for the suffering, beyond stressing the naturalness of all emotions, explaining how the vicious cycle functions for the individual, and highlighting the specific desires underlying fears.

A hypochondriacal temperament is an inclination to be oversensitive to psychological difficulties, physical sensations, and social interactions.

Inadaptability anxiety is Kora’s in-depth reinterpretation of Morita’s concept of the hypochondriacal temperament. Kora presents this concept as the mood in which an introvert feels himself or herself at a disadvantage in relation to his or her mental and physical state; in other words, this is the introvert’s experience of the anxiety regarding his or her inadaptability to his or her environment.

Psychic interaction describes the case where commonplace sensations that were ignored in the past now take on a sudden new meaning, provoking an intense anxiety reaction that cannot be ignored. The mental process or the person’s attention slips out of control of conscious intention, and he or she begins instead to concentrate his or her attention on sensations such as the heart’s throbbing and palpitation and the shortness of the breath due to the sudden anxious reaction to the idea of having a weak heart and being prone to suffer a heart attack at any moment. This reaction arises from a judgment based on a hypochondriacal tendency or temperament. As a result, the receptiveness to such sensations becomes highly sensitized, and this tendency further intensifies sensations and lowers practical functionality. Attention becomes more and more focused on these sensations. This process is what Morita terms psychic interaction.

Contradiction by ideas refers to thinking about what one might be or should be, as opposed to what one actually is. A person with a hypochondriacal temperament affected in this way will self-evaluate his or her true self negatively. Such a person aspires to an ideal self-image that is on a high plane and is inclined to “should” thinking. This aspiration puts the phenomenon of readily denying the fact of what one actually is into play.

Toraware (preoccupation) is a mental fixation, mental attachment or blocked flow of attention and mental energy due to cognitive rigidity and preoccupations with certain aspects of physical and mental experience that restricts the peripheral awareness necessary to respond to current circumstances. In Morita therapeutic theory, toraware is composed of psychic interaction and ideational contradiction. Reducing toraware in Morita therapy is therefore one of the critical turning points in patients’ therapeutic progress.

Arugamama (“as-is” or “as it is”) is a concept that is opposed to toraware and the primary Morita therapeutic goal, in which one accepts reality as it is and responds accordingly. It is not a psychological state as such but the redirection of energy and attention to fulfill daily tasks.
